# Association of preoperative platelet counts with in-hospital mortality in patients with acute type A aortic dissection: a retrospective single-center study

**DOI:** 10.3389/fcvm.2025.1524252

**Published:** 2025-04-23

**Authors:** Yifei Zhou, Wuwei Wang, Quan Liu, Hongwei Jiang, Rui Fan, Xin Chen

**Affiliations:** ^1^ School of Medicine, Southeast University, Nanjing, Jiangsu, China; ^2^ The Department of Thoracic and Cardiovascular Surgery, Nanjing First Hospital, Nanjing Medical University, Nanjing, Jiangsu, China

**Keywords:** platelet, type A aortic dissection, association, mortality, mid-term outcome

## Abstract

**Background:**

This study aimed to investigate the relationship between preoperative platelet (PLT) count levels and postoperative in-hospital death and mid-term survival in patients with acute type A aortic dissection (ATAAD).

**Methods:**

A total of 341 ATAAD patients who underwent surgery between January 2019 and December 2023 were enrolled in this retrospective study. Preoperative PLT count levels were compared between the two groups for whether in-hospital death occurred. Then, patients were divided into two groups according to the optimal cutoff value by the maximum Youden index (184.5), and confounders were adjusted by multiple models to confirm whether low PLT count was an independent risk factor for in-hospital death after surgery. Kaplan–Meier method was used to draw the survival curve for the mid-term follow-up.

**Results:**

Patients who suffered in-hospital death had lower preoperative PLT count levels. After grouping by PLT count, patients in the low PLT count group exhibited higher in-hospital mortality (16.9% vs. 5.5%; *P* = 0.004). Univariate logistic regression analysis indicated that ATAAD patients with low PLT count were prone to death during hospitalization [odds ratio (OR): 4.549; 95% confidence interval (CI): 1.515–13.654, *p* < 0.05]. After adjustment for the potential confounders, low PLT count remained an independent risk factor with postoperative in-hospital death (OR: 3.443, 95%CI: 1.400–8.468, *p* < 0.05). Mid-term follow-up showed that there was a significant difference in overall survival between different PLT count groups (HR: 3.154; 95%CI: 1.495–6.654, *p* < 0.05).

**Conclusion:**

A lower level of preoperative PLT count was an independent risk factor for in-hospital death in patients with ATAAD and had a lower survival rate at mid-term follow-up.

## Introduction

Acute type A aortic dissection (ATAAD) is a highly lethal disease, with mortality increasing by 1% to 2% per hour in the early stages after symptom onset (1). If not treated in time, the overall mortality rate can reach 90% (2), however, even after emergency surgical treatment, the patient mortality can still be up to 25% (3). Given the high mortality rate of such kind of disease, preoperative identification of high-risk patients is critical. Studies on type A dissection have shown that activation of PLT and coagulation/fibrinolytic system plays an important role (4). In addition, PLT activation can aggravate systemic inflammation by inducing the release of proinflammatory mediators from vascular endothelial cells. Currently, PLT/inflammation-related biomarkers such as SII (systemic immune-inflammation index), PLR (PLT to lymphocyte ratio), and so on have shown favorable predictive value in clinical outcomes (5, 6). PLT seems to play a role as a bridge between coagulation and inflammation in type A aortic dissection. Based on this, it is important to explore whether PLTs can identify high-risk patients with acute type A aortic dissection. Low PLT count levels are often associated with adverse events, one previous study showed that low admission PLT counts predict an increased risk of in-hospital mortality in patients with type A acute aortic dissection (7), however, this study had a small sample size and no long-term follow-up. Our study was designed with a relatively large sample size to investigate the relationship between preoperative PLT count level and in-hospital mortality in patients with ATAAD, and the relationship between preoperative PLT count level and mid-term survival rate after operation.

## Methods

### Study population

Patients with acute type A aortic dissection who underwent emergency surgery in our center between January 2019 and December 2023 were retrospectively included. Inclusion criteria: (1) Age ≥18 years old; (2) Stanford type A aortic dissection confirmed by CT angiography (CTA); (3) The onset was within 24 h and received emergency operation. Exclusion criteria: (1) Chronic aortic dissection or more than 24 h from onset to surgery; (2) Previous history of cardiovascular surgery; (3) Iatrogenic aortic dissection during other types of cardiovascular surgery; (4) Complicated with chronic hepatic and renal insufficiency or autoimmune diseases; (5) Traumatic aortic dissection; (6) Incomplete case data. Finally, a total of 341 subjects were included in this study. All patients underwent Sun's operation, namely total aortic arch replacement with frozen elephant trunk operation. The study was approved by the Ethics Committee of Nanjing First Hospital (approval number: KY20220425-05) and complied with the guidelines of the Declaration of Helsinki.

### Data collection

The clinical data of the enrolled patients were retrieved from the electronic medical record system, including baseline characteristics (age, gender, etc.), preoperative chronic history (hypertension, diabetes, coronary heart disease, etc.), and preoperative blood tests; The intraoperative data (cardiopulmonary bypass time, aortic cross-clamp time, etc.) and postoperative clinical data were analyzed. The primary endpoint was in-hospital death and secondary endpoint events for postoperative complications. Follow-up included patients for all-cause death.

### Definition

Postoperative acute kidney injury (PO-AKI) was evaluated in accordance with the Kidney Disease: Improving Global Outcomes (KDIGO) guidelines within 1 week postoperatively, defined by any of the following criteria: an increase in serum creatinine (SCr) of ≥0.3 mg/dl (≥26.5 µmol/L) within 48 h; an increase in SCr to ≥1.5 times the baseline value, known or presumed to have occurred within the preceding 7 days; or a urine output of <0.5 ml/kg/h for 6 h. Preoperative shock was diagnosed with systolic blood pressure <90 mm Hg.

### Surgical approach and techniques

The patient underwent a median sternotomy after general anesthesia, CPB and selective cerebral perfusion were performed using right axillary artery and right atrial cannula. A vent catheter is inserted into the left atrium through the right upper pulmonary vein to decompress the left ventricle. The patient was cooled to a nasopharyngeal temperature of approximately 24°C by CPB. To protect the myocardium, anterograde injection of cold cardioplegia was used. During cooling, the brachiocephalic artery is exposed and surgery is performed on the proximal aorta. When the bladder temperature dropped to 27℃, the right axillary artery cannula continued to infuse the brain at a rate of about 5–10 ml/kg min. If the right axillary artery was not suitable for cannulation, the femoral artery should be cannulated, cerebral perfusion was performed by direct perfusion of the brachiocephalic artery when circulation was stopped. After the distal anastomosis was completed, systemic blood perfusion was restored and the temperature was gradually rewarmed. In the rewarming period, proximal aortic anastomosis and other operations were performed to restore coronary blood flow. All patients were admitted to intensive care unit for routine postoperative monitoring.

### Statistical analysis

All data were analyzed with SPSS version 26 (SPSS Inc., Chicago, IL, USA). Kolmogorov Smirnov was used to test the normality of the distribution of the data. Continuous variables were presented as means ± standard deviations or as medians with interquartile ranges, contingent upon whether they had a normal distribution. Non-paired Student's *t*-test was utilized for normally distributed measurement data, while the Mann–Whitney *U* test was adopted for non-normally distributed measurement data. Categorical variables were expressed as percentages and analyzed through the chi-square test. A *p*-value of less than 0.05 was regarded as statistically significant. To explore the relationship between the preoperative PLT count level and in-hospital mortality, four models were constructed. The crude model was established without adjustment for confounding factors. Model 1 was adjusted for age and sex; Model 2 was adjusted for sex, age, CPB time, APTT (Activated Partial Thromboplastin Time), preoperative shock, preoperative lower extremity ischemia, and concomitant CABG (Coronary Artery Bypass Grafting) procedure; Model 3 was adjusted for sex, age, CPB time, APTT, preoperative shock, concomitant CABG procedure, hypertension, diabetes, preoperative AF, preoperative hemoglobin, preoperative WBC (White Blood Cells), and fibrinogen. For follow-up, the Kaplan-Meier method was used to draw the survival curve, and the log-rank test was performed. The association between platelet count and postoperative mid-term mortality was evaluated by Cox proportional hazards analysis.

## Results

### Baseline characteristics

**Figure 1:**
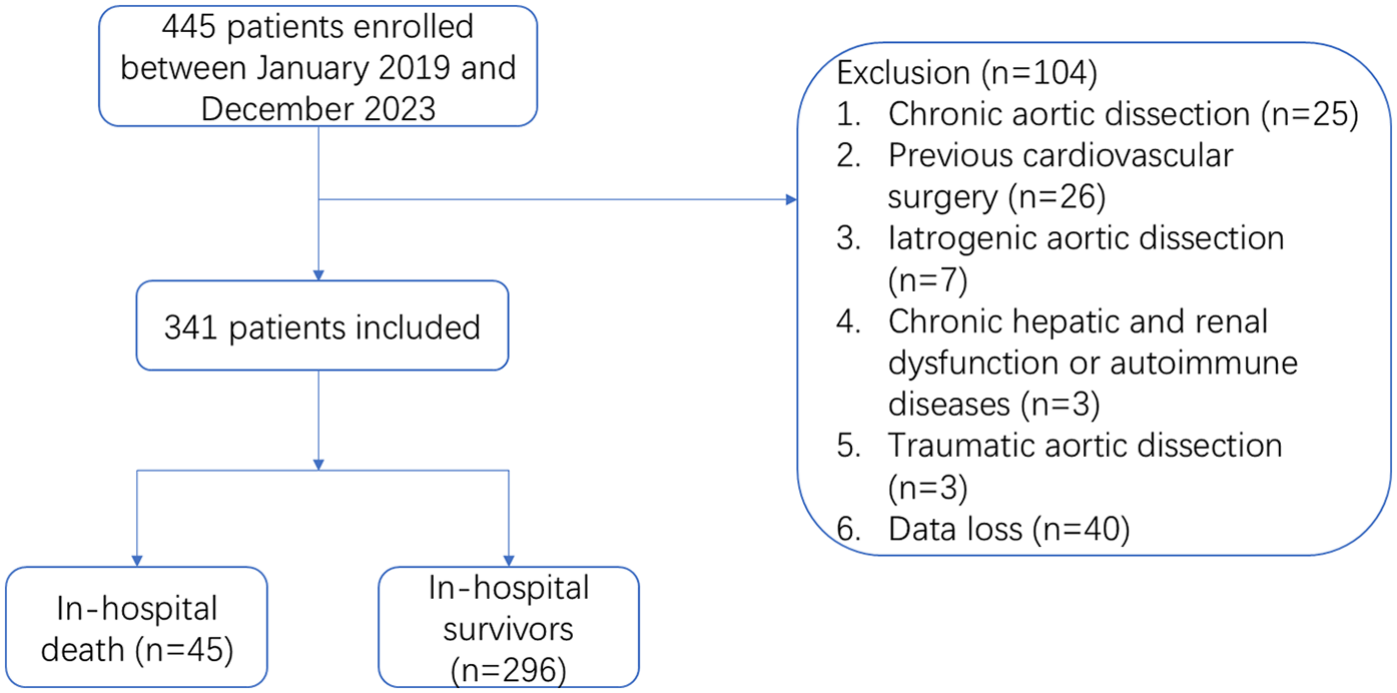
Flow chart of the study.

**Figure 2:**
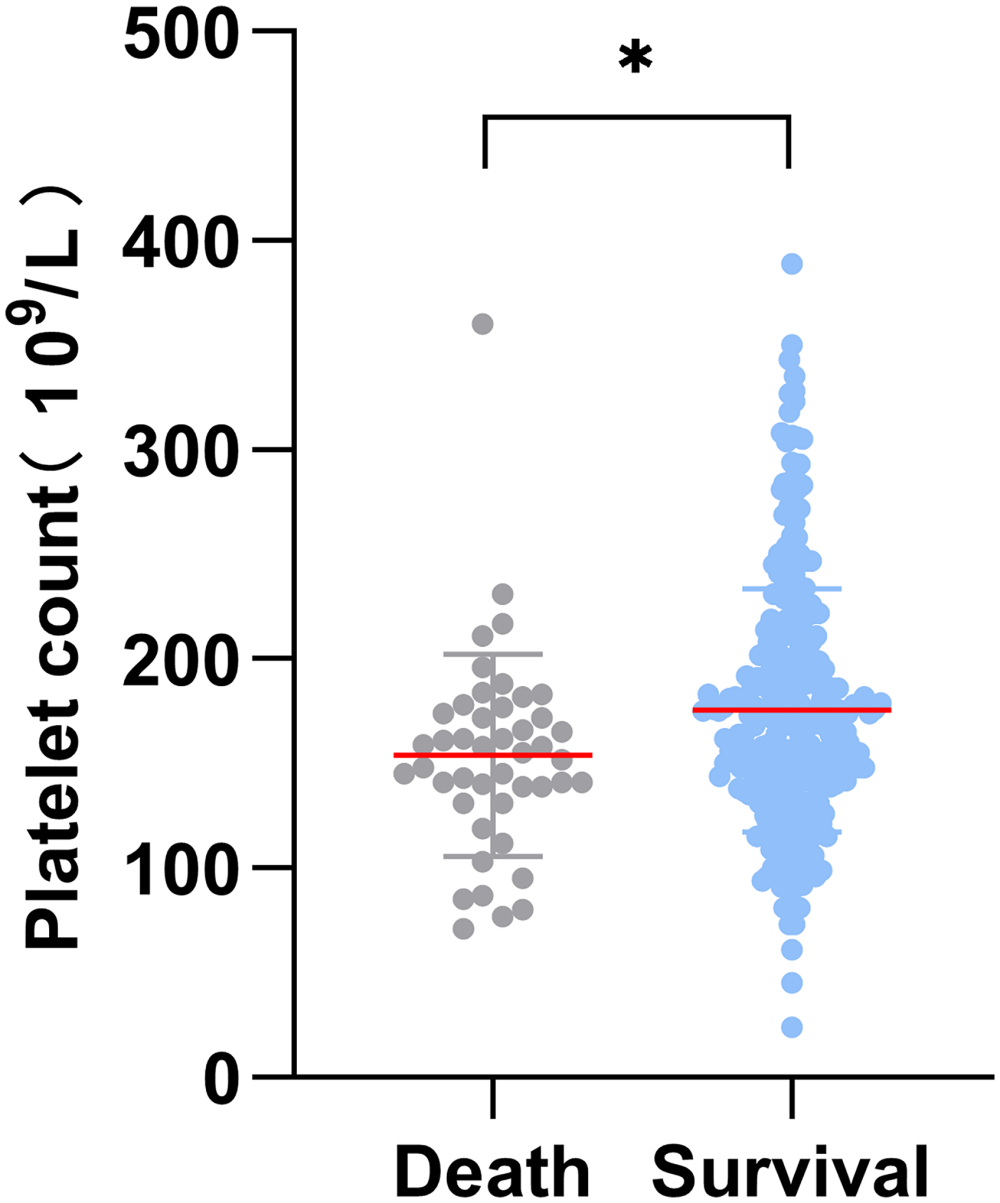
Distribution of preoperative platelet counts between death and survival groups during hospitalization. *P value < 0.05.

**Figure 3:**
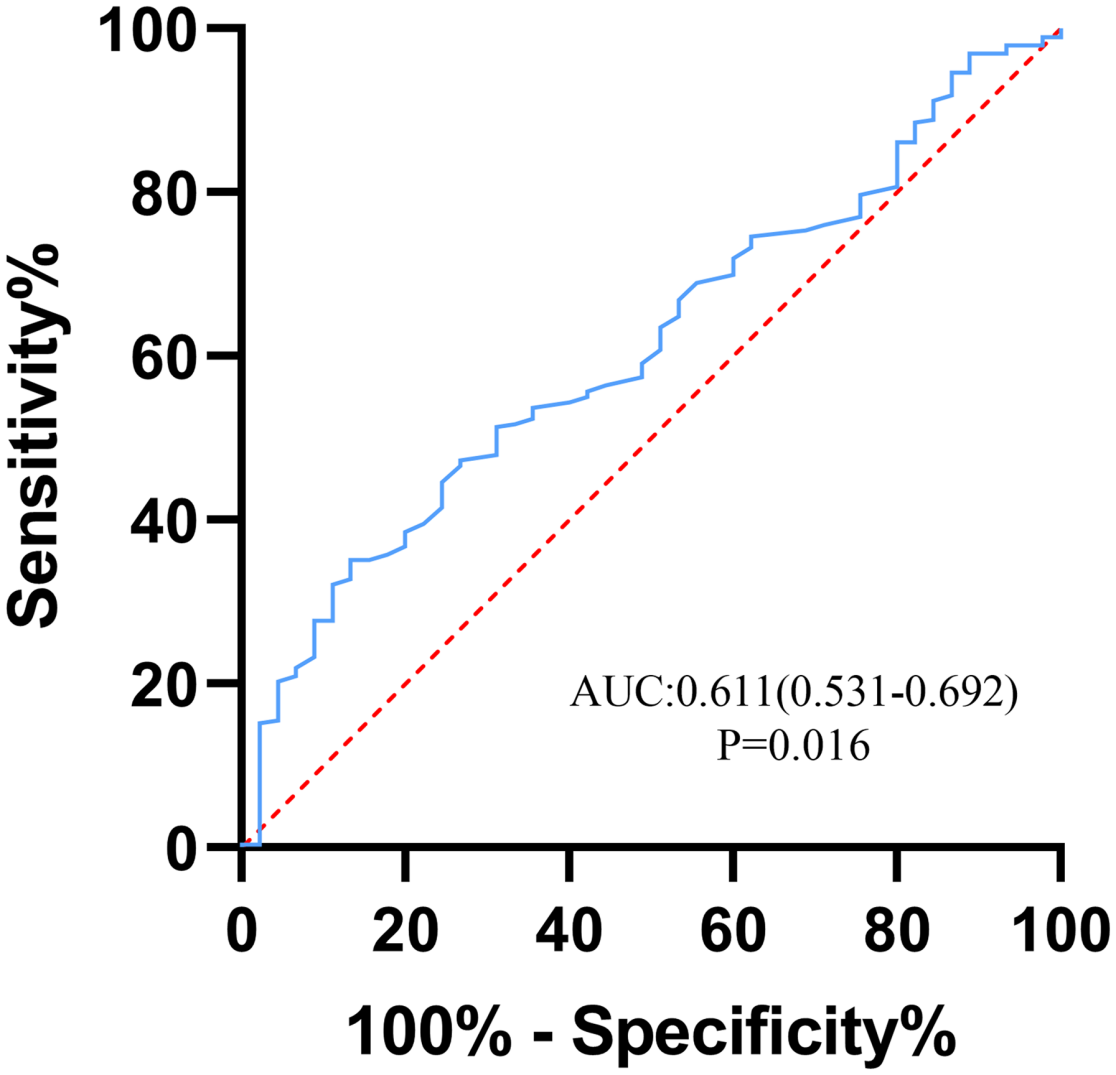
ROC curve of platelet for predicting in-hospital mortality in patients with acute type A aortic dissection. The area under the curve (AUC) was 0.611 (95% CI: 0.531–0.692). ROC, receiver operating characteristic.

A total of 445 patients from January 2019 to December 2023 were included in the study, excluding 40 patients with incomplete medical records, 25 patients with chronic aortic dissection, 26 patients with cardiovascular surgery history, 7 patients with iatrogenic aortic dissection, 3 patients with chronic hepatic and renal insufficiency or autoimmune diseases, and 3 patients with traumatic aortic dissection. 341 subjects were finally included in the study (Figure 1). Among them, 45 (13.2%) occurred in-hospital deaths, there were 254 males (74.5%) and 87 females (25.5%) in this study. The average age of the patients was 55.0 ± 12.6. The preoperative PLT count in the death group was significantly lower than that in the survival group [(154.1 ± 48.3) vs. (175.5 ± 58.2), *p* < 0.05] (Figure 2). To classify patients with ATAAD into high-platelet and low-platelet groups, a new cutoff value needs to be determined. We used the maximum Youden index (sensitivity + specificity −1) based on the ROC curve (AUC: 0.611, 95%CI: 0.531–0.692) (Figure 3) to calculate the optimal cutoff value, and finally, 184.5 was confirmed as the cutoff value (sensitivity: 0.867, specificity: 0.351).

### Preoperative and intraoperative clinical characteristics of different PLT groups

As shown in Table 1. In the H-PLT group, the preoperative hemoglobin, white blood cell count, and fibrinogen levels were significantly higher than those in the L-PLT group (*p* < 0.05), while the APTT level between the two groups was the opposite, and the difference was statistically significant. There was no significant difference in variables such as age, gender, and preoperative chronic history (hypertension, diabetes, etc.). Intraoperative variables such as combined CABG surgery, CPB time, and aortic cross-clamp time, between the two groups of patients, were no significant statistical differences.

**Table 1:** Demographic and laboratory examination of patients with acute type A aortic dissection grouped according to preoperative platelet count levels.

Variables	L-PLT (PLT <184.5 *n* = 231)	H-PLT (PLT ≥184.5 *n* = 110)	*P* value
Age, y	55.5 ± 12.6	54.0 ± 12.7	0.303
Male, *n* (%)	174.0 (75.3)	80.0 (72.7)	0.607
BMI, kg/m^2^	26.2 ± 4.1	26.8 ± 4.1	0.225
Smoking history, *n* (%)	122 (52.8)	53 (48.2)	0.424
Drinking history, *n* (%)	72 (31.2)	35 (31.8)	0.904
Hypertension, *n* (%)	189 (81.8)	91 (82.7)	0.838
Diabetes, *n* (%)	21 (9.1)	13 (11.8)	0.432
Hyperlipidemia, *n* (%)	12 (5.2)	1 (0.9)	0.053
Dialysis, *n* (%)	3 (1.3)	0	0.230
COPD, *n* (%)	7 (3.0)	1 (0.9)	0.755
Cerebral infarction history, *n* (%)	13 (5.6)	5 (4.5)	0.676
Coronary artery disease, *n* (%)	20 (8.7)	9 (8.2)	0.883
Preoperative shock, *n* (%)	10 (4.3)	5 (4.5)	0.927
Preoperative cardiopulmonary resuscitation, *n* (%)	4 (1.7)	1 (0.9)	0.555
Preoperative AF, *n* (%)	5 (2.2)	1 (0.9)	0.410
Lower extremity ischemia, *n* (%)	52 (22.5)	24 (21.8)	0.886
Preoperative hemoglobin (g/L)	130.8 ± 18.8	135.3 ± 18.9	0.039*
PT (s)	12.2 (9.9–32.8)	12.0 (9.7–17.8)	0.231
Preoperative WBC (10^9^/L)	12.7 ± 3.9	14.2 ± 4.6	0.001*
Fibrinogen (g/L)	2.4 ± 1.3	3.2 ± 1.8	<0.001*
APTT (s)	29.4 ± 5.1	27.7 ± 4.3	0.003*
Urea nitrogen (mmol/L)	7.1 ± 3.1	6.5 ± 2.1	0.083
Creatinine (μmol/L)	79.8 (59.4–105.5)	79.25 (63.4–107.4)	0.197
Interval hours between onset of symptoms and arrival time in ER (h)	3.5 ± 1.0	3.4 ± 1.1	0.593
Concomitant CABG procedures, *n* (%)	24 (10.4)	10 (9.1)	0.708
CPB time (min)	171.0 (147.0–195.0)	171.5 (148.8–193.8)	0.489
Aortic cross-clamp time (min)	93.0 (81.0–111.0)	95.0 (77.0–110.5)	0.778

BMI, body mass index; COPD, chronic obstructive pulmonary disease; PT, prothrombin time; AF, atrial fibrillation; WBC, white blood cells; APTT, activated partial thromboplastin time; CPB, cardiopulmonary bypass; ER, emergency room.

* *P* < 0.05.

### Postoperative clinical outcome between different PLT groups

As depicted in Table 2, patients with ATAAD in the low PLT group (<184.5) exhibited higher in-hospital mortality (16.9% vs. 5.5%; *P* = 0.004). There were no significant differences in the incidence of postoperative acute renal insufficiency, gastrointestinal bleeding, paraplegia, prolonged mechanical ventilation, or secondary tracheal intubation between the two groups (*p* > 0.05).

**Table 2:** Differences in postoperative adverse events between the two groups.

Variables	L-PLT (*n* = 231)	H-PLT (*n* = 110)	*P* value
In-hospital death	39 (16.9)	6 (5.5)	0.004*
AKI	55 (23.8)	17 (15.5)	0.077
Reintubation	20 (8.7)	10 (9.1)	0.895
Prolonged mechanical ventilation	111 (48.1)	46 (41.8)	0.280
Paraplegia	8 (3.5)	4 (3.6)	0.935
Gastrointestinal bleeding	10 (4.3)	8 (7.3)	0.256

AKI, acute kidney injury.

* *P* < 0.05.

### Associations between PLT and in-hospital death

Univariate logistic regression analysis was conducted on ATAAD patients, and the preoperative PLT count was found as the risk factor for in-hospital death (OR: 4.549, 95%CI: 1.515–13.654, *p* = 0.007). After adjusting for confounding factors, the effect of PLT on patient death during hospitalization was elucidated through the adjusted models (Table 3). After multivariable adjustment, the results still suggested that preoperative low PLT count was a significant risk factor for in-hospital mortality in ATAAD patients after surgery, with model 1 (OR: 4.450, 95%CI: 1.528–12.957, *p* = 0.006), model 2 (OR: 3.599, 95%CI: 1.371–9.449, *p* = 0.009), model 3 (OR: 3.443, 95%CI: 1.400–8.468, *p* = 0.007).

**Table 3:** Analysis of risk factors for in-hospital death by adjusting confounder models.

In-hospital Death	H-PLT	L-PLT	*P* value
Unadjusted	Ref	4.549 (1.515–13.654)	0.007*
Model 1	Ref	4.450 (1.528–12.957)	0.006*
Model 2	Ref	3.599 (1.371–9.449)	0.009*
Model 3	Ref	3.443 (1.400–8.468)	0.007*

Model 1: adjust for sex and age.

Model 2: adjust for sex, age, CPB time, APTT, preoperative shock, preoperative lower extremity ischemia, and CABG.

Model 3: adjust for sex, age, CPB time, APTT, preoperative shock, CABG, hypertension, diabetes, preoperative AF, preoperative hemoglobin, preoperative WBC, and fibrinogen.

CPB, cardiopulmonary bypass; APTT, activated partial thromboplastin time; CABG, coronary artery bypass grafting.

* *P* < 0.05.

### Mid-term outcomes

**Figure 4:**
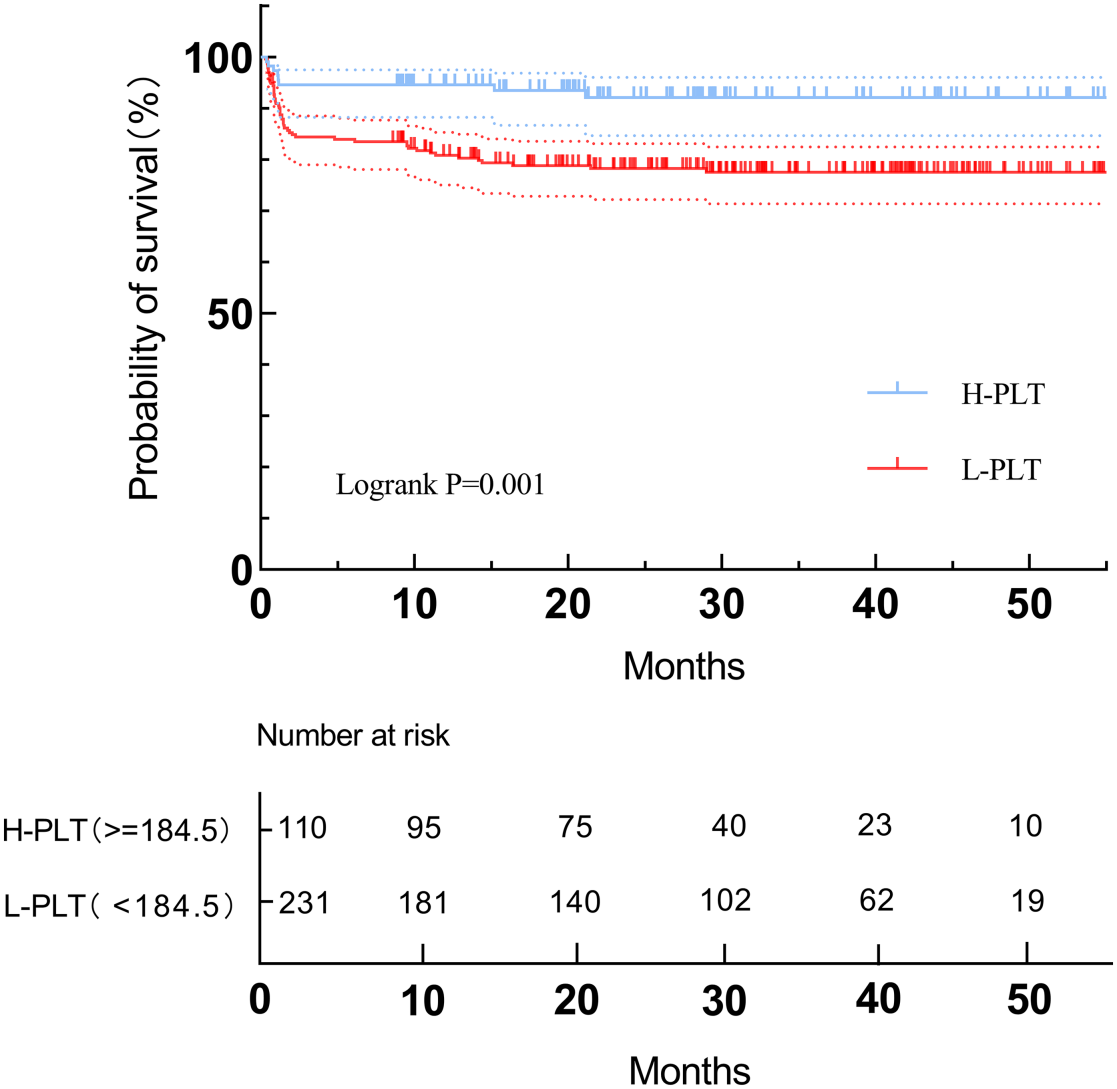
Survival curve analysis of time trend of mortality in postoperative follow-up.

Midterm follow-up of survival was 100% complete. Patients were followed up for a median period of 2.2 years. A total of 8 deaths occurred in the H-PLT group while 50 occurred in the L-PLT group. For mid-term mortality, patients in the low PLT group had a higher mortality rate than those in the high PLT group, and the difference was statistically significant. Kaplan-Meier curve (Figure 4) also showed that there was a significant difference in overall survival between the two groups (Log-rank *p* = 0.001). In addition, Cox proportional hazard analysis results suggested that patients with low platelet levels, as a categorical variable, had a higher risk of mid-term death (HR: 3.154, 95%CI 1.495–6.654, *P* = 0.003).

## Discussion

Acute type A aortic dissection is considered to be a major threat to people's health due to its high mortality. Therefore, early identification of high-risk patients can effectively reduce the risk of death. This study aims to investigate the relationship between preoperative PLT count and in-hospital mortality in patients with acute type A aortic dissection undergoing surgical treatment. The results showed that patients with lower PLT count levels (<184.5 × 10^9^/L) at admission had a higher risk of death during hospitalization. After adjusting for confounding factors, lower PLT count level was still an independent risk factor for in-hospital mortality. In the results of the mid-term follow-up, the survival rate of the low PLT group was significantly lower than that of the high PLT group. These results suggest that preoperative PLT count level can be used as a new biological indicator to identify high-risk patients with acute type A aortic dissection.

PLTs originate from megakaryocytes, and their primary functions encompass coagulation, hemostasis, and the repair of damaged blood vessels. Nevertheless, recent investigations on PLTs have demonstrated that they also exert a crucial role in inflammation and immunity. It is widely acknowledged that type A aortic dissection is intimately associated with inflammation, and coagulation dysfunction has likewise been indicated to be closely related to type A aortic dissection (8). The study conducted by Sbarouni et al. suggested that inflammation-induced thrombosis within the false lumen of aortic dissection can facilitate PLT activation, further accelerating PLT consumption and ultimately influencing the prognosis of patients (9). Additionally, inflammation can modify PLT reactivity in patients with thrombocytopenia undergoing percutaneous coronary intervention (10). PLTs can also reprogram monocyte functions by secreting MMP-9 (11), which occurs and plays an important role in type A aortic dissection development (12). This turns PLTs into a bridge linking inflammation and coagulation. These studies imply that PLTs play a significant role in type A aortic dissection.

So far, there are novel hematological biomarkers that play a prognostic role in aortic dissection, such as the significant association between neutrophil to lymphocyte ratio and in-hospital mortality (13). PLT count level has been shown to have good predictive value for postoperative adverse events in cardiovascular diseases, such as in a study of elderly people undergoing valve replacement surgery suggesting that postoperative low PLT count levels were strongly associated with in-hospital death (14). In addition, the level of PLT also has good prognostic value in patients with aneurysmal subarachnoid hemorrhage (15). Besides, in a model for ischemic stroke after dissection, PLT count levels were inversely associated with the incidence of outcome events (16). It is worth noting that the cutoff value obtained in this study is not particularly low, which may be because it takes a certain amount of time for platelet activation to reach the highest level. It was mentioned in a previous study that platelet activation reaches the highest level 4 h after surgery (17), but the deeper reasons need to be further explored. Platelet-related indicators such as platelet-lymphocyte ratio can predict the limb survival in critical limb ischemia (18).

The mechanism for the correlation between reduced PLT and aortic dissection is unclear. There are several possible explanations for the association of PLTs with in-hospital mortality. Firstly, when aortic dissection occurs, the vascular endothelial injury will promote PLT aggregation, which will lead to abnormalities in the systemic coagulation system and cause an increase in markers for activation of coagulation (8, 19). Lower fibrinogen levels in the low platelet group may result in a higher disseminated intravascular coagulation (DIC) status, and preoperative DIC has been reported to be associated with a poorer prognosis (20). Secondly, the occurrence of type A aortic dissection is related to the inflammatory response, which aggravates the necrosis and apoptosis of smooth muscle cells, causes the degradation of elastic tissue, and finally causes aortic dissection. The inflammatory response of the blood vessels further activates the PLTs, causing them to become activated and adhere to the torn vessel wall. Activated PLTs can release inflammatory cells recruited from PLT granules and interact with them to further aggravate vascular inflammation and cause dissection injury (21). In recent years, the significant role of neutrophil extracellular traps (NETs) in the inflammatory response has been increasingly emphasized. According to new research, NETs have been implicated in numerous cardiovascular diseases (22–24). PLTs also play an important role in the production of NETs and tissue injury. PLT interaction with activated neutrophils is a potent inducer of NETs (25). NETs have also been proven highly expressed in aortic dissection (26), which shows the PLT-related inflammatory response in the aortic dissection. To sum up, the low PLT count level suggests a relatively high coagulopathy condition and a high inflammatory response, which is also consistent with the progression of type A aortic dissection. Furthermore, the high expression of some factors released by PLTs such as PLT-derived growth factor B (PDGF-B) and Thrombospondin 1 (TSP1) in type A aortic dissection may also indicate the role of PLTs in the disease (27, 28). Moreover, in a study of preoperative anti-PLT therapy in patients with aortic dissection, it was shown that patients treated with preoperative anti-PLT therapy had more intraoperative blood transfusion and postoperative mortality (29), which is consistent with our findings. However, these possible causes are currently hypothesized, and further research is needed to confirm the specific mechanisms involved.

As a more routine measurement item in clinical detection, PLT count is easy to obtain. Considering the predictive value of PLT count level for in-hospital death and mid-term survival in patients with acute type A aortic dissection, in clinical practice, this index can be used to comprehensively reflect the coagulation and inflammation levels of patients, to be an important supplement to identify high-risk patients and prognostic indicators of acute type A aortic dissection before surgery, which has important clinical significance.

However, there are some limitations in our study. Firstly, this study is a single-center retrospective study, and further studies with large samples from multiple centers are needed to verify it. Secondly, for patients with low preoperative PLT count levels, how to effectively manage the perioperative period, such as the amount of PLT transfusion, deserves further research. Finally, because of the limited follow-up time in this study, the long-term adverse events of patients need to be further studied. In addition, there is likely to be a depletion of platelets in the interval between onset of symptoms and arrival time at the emergency room of the dissection, which will affect this study and require further studies to prove.

## Conclusion

Our research demonstrates that preoperative PLT <184.5 constitutes an independent risk factor for in-hospital mortality. As a simple and feasible evaluation index, PLT count possesses a certain association with in-hospital mortality in ATAAD patients undergoing surgery, implying that ATAAD patients with low PLT count should receive more attention.

## Data Availability

The raw data supporting the conclusions of this article will be made available by the authors, without undue reservation.
